# Rapid Antibiotic Susceptibility Testing of Gram-Negative Bacteria Directly from Urine Samples of UTI Patients Using MALDI-TOF MS

**DOI:** 10.3390/antibiotics12061042

**Published:** 2023-06-12

**Authors:** Felix R. Neuenschwander, Birgit Groß, Sören Schubert

**Affiliations:** Max von Pettenkofer Institute of Hygiene and Medical Microbiology, Faculty of Medicine, LMU Munich, Elisabeth-Winterhalter-Weg 6, 81377 Munich, Germany; neuenschwander@mvp.lmu.de (F.R.N.); gross@mvp.lmu.de (B.G.)

**Keywords:** urinary tract infection, antimicrobial resistance, MALDI-TOF MS, rapid AST, antibiotics

## Abstract

Urinary tract infections (UTIs) are one of the most common human infections and are most often caused by Gram-negative bacteria such as *Escherichia coli*. In view of the increasing number of antibiotic-resistant isolates, rapidly initiating effective antibiotic therapy is essential. Therefore, a faster antibiotic susceptibility test (AST) is desirable. The MALDI-TOF MS-based phenotypic antibiotic susceptibility test (MALDI AST) has been used in blood culture diagnostics to rapidly detect antibiotic susceptibility. This study demonstrates for the first time that MALDI AST can be used to rapidly determine antibiotic susceptibility in UTIs directly from patients’ urine samples. MALDI-TOF MS enables the rapid identification and AST of Gram-negative UTIs within 4.5 h of receiving urine samples. Six urinary tract infection antibiotics, including ciprofloxacin, cotrimoxazole, fosfomycin, meropenem, cefuroxime, and nitrofurantoin, were analyzed and compared with conventional culture-based AST methods. A total of 105 urine samples from UTI patients contained bacterial isolates for MALDI AST. The combination of ID and AST by MALDI-TOF allowed us to interpret the result according to EUCAST guidelines. An overall agreement of 94.7% was found between MALDI AST and conventional AST for the urinary tract pathogens tested.

## 1. Introduction

**Figure 1:**
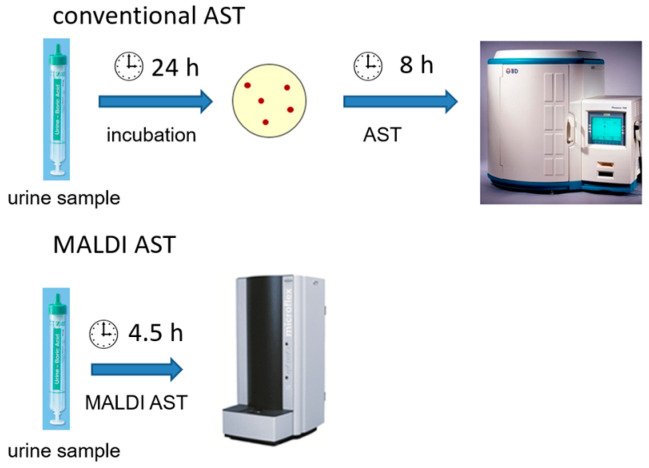
Comparison of the time-to-results between conventional antimicrobial susceptibility testing (AST) and matrix-assisted laser desorption/ionization-based AST (MALDI AST).

Urinary tract infections (UTIs) are among the most common infections, affecting approximately 150 million people worldwide each year [1]. In the US alone, 400,000 patients were hospitalized because of UTIs in 2011, at a cost of approximately USD 2.8 billion [2]. The risk factors for UTIs include being female [3,4], immunosuppression, diabetes mellitus [5], and indwelling catheters. Therefore, UTIs are also a common cause of nosocomial infections [6,7,8]. *E. coli* is by far the most frequently isolated pathogen of UTIs [1,9] and often causes complications such as urosepsis. Other Gram-negative bacteria (*Enterobacterales*) originating from the intestine are also frequently found to be causative agents of UTIs [10]. With a growing number of Gram-negative bacteria showing antibiotic resistance [11], it is desirable to obtain antimicrobial susceptibility testing (AST) results much sooner than the usual 48 h after sample collection. Same-day AST results could allow for the timely adjustment of antibiotic therapies or even the initiation of targeted therapy. This could avoid ineffective antibiotic therapy and the selection of resistant pathogens. Matrix-assisted laser desorption/ionization time-of-flight mass spectrometry (MALDI-TOF MS) is the standard for bacterial identification in microbiological diagnostic laboratories [12,13]. Since this technology is now widely used in microbiological laboratories, rapid AST using MALDI TOF MS represents a good opportunity to apply this method for further diagnostics without limiting its use to specialized laboratories. In the present study, the MALDI Biotyper antibiotic susceptibility test rapid assay (MBT ASTRA Assay) [14], which is a MALDI-TOF MS-based rapid phenotypic AST method (MALDI AST), was extended to a new area of application in rapid ASTs for UTI pathogens directly on urine samples. Because Gram-negative bacteria account for the vast majority of UTIs, this study focuses on these pathogens. In this proof-of-concept study, we demonstrate that the MALDI AST enables antibiotic susceptibility test results for UTI bacteria directly from urine samples in as little as 4.5 h. In contrast, it takes 48 h to obtain results with conventional culture-based microbiological diagnostics (Figure 1). To verify the MALDI AST results, bacteria from 105 urine samples from UTI patients were compared with conventional ASTs, using the minimum inhibitory concentration (MIC)-based AST as the gold standard. MICs were interpreted according to clinical breakpoints from the European Committee on Antimicrobial Susceptibility Testing (EUCAST) [15].

## 2. Results

The study consists of two consecutive parts. In the first part of the study, the samples suitable for the MALDI AST were selected from all the urine samples received. This was necessary because the majority of urine samples showed no bacterial growth or urethral flora growth with a low bacterial count. As described in the methods section of this study, the urine samples were screened using dipstick testing and flow cytometry. Therefore, pyuria and nitrite positivity or the Gram-negative flag served as surrogate markers for *Enterobacterales*. Several studies have demonstrated that the UF 5000 shows a good correlation in white blood cell (WBC) counts with microscopy [16] and the Gram-negative flag with culturally found pathogens [17,18,19], making the UF 5000 a proven method of screening urine samples. In total, the screening resulted in 198 suitable urine specimens for the MALDI AST.

**Figure 2:**
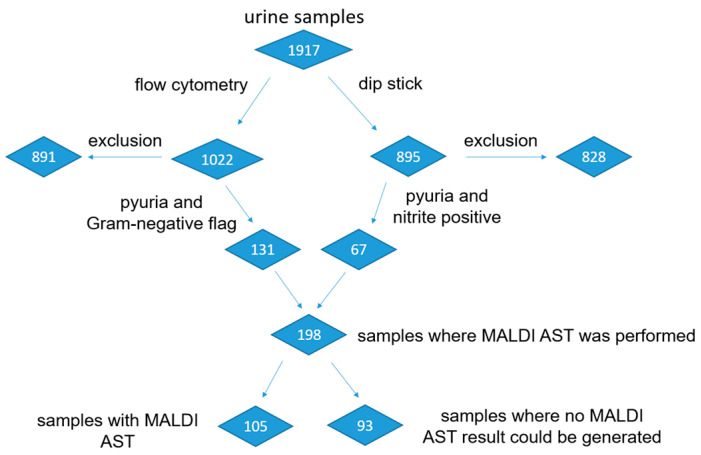
Flowchart of the urine samples in the screening process and the matrix-assisted laser desorption/ionization antimicrobial susceptibility testing (MALDI AST) assay.

In the second part of the study, MALDI ASTs were performed directly on patient urine samples and compared with a conventional MIC-based AST. Figure 2 provides an overview of all screened urine samples and the respective MALDI AST results collected in this study. In total, MALDI AST results were successfully obtained from 105 urine samples, of which 95 had monomicrobial and 10 had polymicrobial infections. Urine samples with polymicrobial infections revealed more than one *Enterobacterales* species. A MALDI AST result could only be generated if a bacterial isolate incubated without antibiotics showed sufficient growth, as defined by protein detection using MALDI-TOF MS. Table 1 provides an overview of the specimens isolated from the urine samples with a successful MALDI AST result. However, 93 urine specimens provided no results using the MALDI AST. Of these urine samples, 69 showed either no bacterial growth in culture, no growth with *Enterobacterales*, a too-low bacterial count, or growth inhibitors such as antibiotics in the urine. No such explanation could be found for 24 isolates.

To evaluate the rapid MALDI AST, only bacteria species with EUCAST clinical breakpoints for the respective antibiotics were included. Below are the MALDI AST results for each antibiotic tested. The MALDI AST enables differentiation between susceptible and resistant isolates. Since no MIC values were obtained, classification as “susceptible, increased exposure” (“I”) was not possible.

Uropathogens from 95 urine specimens were tested for resistance to ciprofloxacin using the MALDI AST assay. Among these, 12 pathogens that showed resistance in the conventional AST were consistently determined to be resistant by the MALDI AST, resulting in a 100% rate of correctly classified resistant isolates. One of the isolates that tested resistant in the MALDI-TOF MS showed a MIC value of 0.38 mg/L using conventional tests with a MIC strip test. The latter result corresponds to the “I” phenotype according to the EUCAST classification. This was included in the evaluation as susceptible. MIC testing identified 83 pathogens as susceptible. Using the MALDI AST, 79 of these were classified as susceptible. This resulted in a 95.2% rate of correctly classified susceptible isolates. Four isolates were incorrectly classified as resistant. In addition to the isolate tested as “I”, two isolates showed MIC values of 0.19 mg/L and 0.064 mg/L.

**Figure 3:**
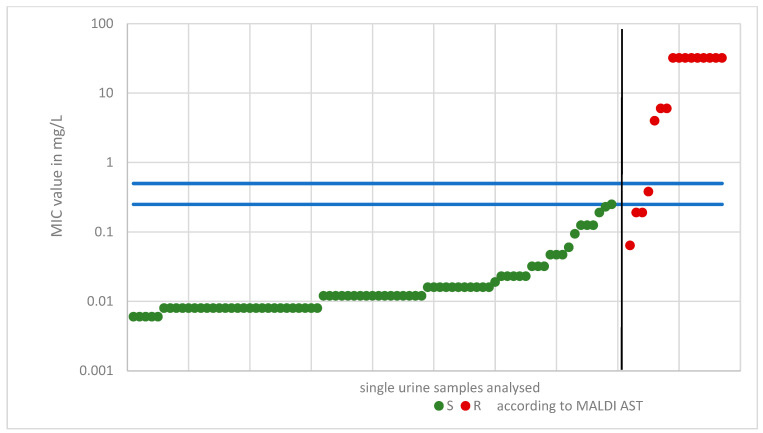
Ciprofloxacin results: Each bacteria sample from a patient’s urine (on the X-axis) is assigned a respective minimum inhibitory concentration (MIC) value from the MIC test strip (on the Y-axis). Each dot represents one sample. All samples are grouped by matrix-assisted laser desorption/ionization antimicrobial susceptibility testing (MALDI AST) results (S, R) and ascending MIC values. Green dots indicate a susceptible result and red dots indicate a resistant result in the MALDI AST assay (separated by the black line). The MIC value breakpoints are 0.25 mg/L and 0.5 mg/L (blue lines).

The ciprofloxacin results are presented in Figure 3. Corresponding figures for the other antibiotics can be found in the Supplementary Materials (Figures S1–S5).

A total of 92 bacteria from patient urine samples were tested for resistance to cotrimoxazole. All 15 pathogens resistant to cotrimoxazole in the MIC determination were also classified as resistant by the MALDI AST, resulting in a 100% rate of correctly classified resistant isolates. Overall, 6 out of 76 cotrimoxazole-susceptible pathogens were incorrectly identified as resistant. This corresponds to a 92.1% rate of correctly classified susceptible isolates. Four *E. coli* isolates, one *Klebsiella pneumoniae* isolate, and one *Enterobacter cloacae* isolate were misclassified. Of these false-resistant classified isolates, four showed relatively high MIC values of 0.75 to 1 mg/L compared with the other susceptible isolates. 

A total of 95 uropathogens from urine samples were tested for resistance to meropenem using the MALDI AST. All pathogens were classified as susceptible, which was confirmed via MIC determination. This resulted in a 100% rate of correctly categorized susceptible isolates. No meropenem-resistant isolates were found in any of the tested urine samples; therefore, no rate of correctly classified resistant isolates could be determined.

For certain bacteria, resistance testing according to the EUCAST clinical breakpoints is only possible for a subset of the tested antibiotics. For nitrofurantoin and fosfomycin, clinical breakpoints are available only for *E. coli*. For cefuroxime, EUCAST breakpoints are provided only for *E. coli*, *Klebsiella* spp. (except *K. aerogenes*), *Raoultella* spp., and *Proteus mirabilis*. 

For cefuroxime, 11 pathogens were determined to be resistant using MIC values, of which 8 were classified as resistant by the MALDI AST, resulting in a 72.7% rate of correctly classified resistant isolates. False-susceptible results were obtained for three *E. coli* isolates. For these isolates, the MIC values determined by the MIC strip test were 24 mg/L for one isolate and 12 mg/L for the others. The latter MIC value is just above the EUCAST clinical breakpoint for cefuroxime: 8 mg/L. Of the 68 pathogens with MIC values below the breakpoint, 55 were also classified as susceptible by the MALDI AST, resulting in an 80.9% rate of correctly classified susceptible isolates. In 13 cases, the results were false-resistant, with two isolates revealing a MIC value of 8 mg/L. These false-resistant results affected nine *E. coli*, three *Klebsiella oxytoca*, and one *Proteus mirabilis* isolate, respectively. 

A total of 66 *E. coli* isolates from urine samples were tested for fosfomycin resistance. All isolates were classified as susceptible by the MALDI AST. These results were fully consistent with conventional ASTs, with the highest MIC value being 24 mg/L. Thus, the rate of correctly classified susceptible isolates was 100%.

The EUCAST guideline provides a nitrofurantoin breakpoint only for *E. coli* from the urine samples of UTI patients. In 66 *E. coli*-positive urine samples, only 1 *E. coli* isolate could be identified as nitrofurantoin-resistant by both the conventional AST and the MALDI AST. The other 65 isolates were classified as susceptible to nitrofurantoin by both methods.

For a complete overview of the pathogens isolated from monomicrobial urine samples and the MALDI AST results, see Table S6 in the Supplementary Materials.

For polymicrobial urine samples, the goal was to identify any resistant pathogens in the sample using MALDI AST. These isolates should show growth in the presence of the antibiotic, thus assessing the whole sample rather than the respective single isolate. A total of 10 urine samples with mixed cultures were tested. In nine cases, two or three fast-growing Gram-negative bacteria were present in equal numbers at 10^5^ CFU/mL. For ciprofloxacin, the presence of one resistant and one susceptible pathogen resulted in a susceptible determination by the MALDI AST. In addition, in one case, there was a false-resistant determination despite the presence of two susceptible pathogens. In the case of cefuroxime, there were four false-resistant determinations despite the presence of two susceptible pathogens. Two samples contained both a cefuroxime-resistant and cefuroxime-susceptible isolate, resulting in a resistant MALDI AST result in one case and a susceptible one in the other. The combination of a resistant and a susceptible pathogen did not occur for cotrimoxazole and meropenem. Since EUCAST clinical breakpoints for nitrofurantoin and fosfomycin are only available for *E. coli* [15], no statement can be made about these non-*E. coli* isolates. In summary, the MALDI AST provided inconclusive results for the polymicrobial urine samples since resistant isolates in these samples could not always be identified as such, and, in some cases, mixtures of susceptible isolates were incorrectly classified as resistant. For a complete overview of the pathogens isolated from polymicrobial urine samples and the MALDI AST results, see Table S7 in the Supplementary Materials.

Overall, for the monomicrobial samples, a general agreement between the conventional AST and the MALD AST was found in 94.7% of all 493 ASTs performed, with major errors (MEs) occurring in only 3 cases and minor errors (MiEs) in 22 cases (Table 2). In the present study, false-resistant isolates are classified as minor errors, and false-susceptible isolates as major errors.

In summary, for the vast majority of bacterium–drug combinations, correct identification and a valid MALDI AST result were obtained within 4.5 h after the samples were submitted to the laboratory. This resulted in significant time-saving compared with conventional AST methods, where AST results are only provided 48 h after the urine samples are received (ME: major error; MiE: minor error).

## 3. Discussion

To our best knowledge, this study shows for the first time that AST results can be obtained directly from a patient’s urine sample with an incubation time of 2.5 h using the MALDI-TOF MS. The results of this phenotypic test can be provided to a clinical physician only about 4.5 h after receiving the sample. 

There are several new rapid genotypic methods for detecting the molecular markers of antibiotic resistance [20,21]. These tests provide very fast results for specific known resistance mechanisms. However, the molecular detection of resistance determinants is associated with a number of uncertainties and disadvantages. (i) There are often a large number of resistance genes, e.g., in the case of beta-lactamases, which differ slightly in the sequence of their coding genes. On the one hand, test systems are not available for all resistance genes; on the other hand, variations in the target sections of the genes can lead to false negative results. (ii) Detecting a gene does not necessarily mean that this gene is also functionally expressed and causes a phenotype. This can lead to false positive results. (iii) Some mechanisms responsible for individual forms of resistance cannot be detected or can only be detected with great difficulty using molecular methods. These include differences in the expression of efflux pump systems and the lower expression of porin proteins in the cell walls of Gram-negative bacteria. The expression of a resistance phenotype is often made up of the sum of individual different resistance determinants. In summary, although molecular resistance gene detection can be used quickly and sensibly for selected resistances (e.g., MRSA, VRE), it is inferior to phenotypic detection and can in no way replace it. Therefore, these tests are a supplemental examination for existing phenotypic AST methods that can provide ASTs for basically all antibiotics regardless of resistance mechanisms. A major disadvantage of phenotypic AST methods is the relatively long result wait period because of the incubation time required to detect bacteria. Recently, various rapid phenotypic AST assays have been developed.

Baltekin et al. [22] showed that antibiotic resistance can be determined within 30 min using direct single-cell imaging. This method is intended to be used in point-of-care diagnostics directly from patient urine samples. At the same time, the bacteria count can be estimated. However, since bacterial identification is not possible with this system, additional testing is required to determine the correct bacteria species and interpret the AST results according to EUCAST species-specific clinical breakpoints. Other novel AST microfluidic systems are designed to determine MIC values within a maximum measurement time of 5 h [23,24,25]. Both described AST methods require investment in additional equipment, which, in addition to the cost of the test panels, also increases the cost of the tests. 

Other studies have investigated the possibility of using flow cytometry for rapid phenotypic ASTs directly on patient urine samples. Toosky et al. [26] demonstrated this method for ASTs on *Enterobacterales* and Gram-positive bacteria. However, as with the method mentioned above, it is not possible to identify the bacteria, which leads to similar problems. In addition, certain antibiotics, such as nitrofurantoin, can interfere with the laser, resulting in apparent reduced bacterial growth [27]. 

In the present study, false-resistant isolates were classified as minor errors and false-susceptible isolates as major errors. While a minor error could tempt the physician not to use a certain effective antibiotic, a major error could lead to incorrect therapy and possibly selecting resistant pathogens. It is important to note that the MALDI AST cannot provide MIC values. 

Overall, we found good agreement between the MALDI AST and the conventional AST using MIC test strips. Meropenem and nitrofurantoin showed no misclassified isolates, while six minor errors (false-resistant) were observed for cotrimoxazole and four for ciprofloxacin. All isolates with false-resistant results for ciprofloxacin and four for cotrimoxazole showed MIC values close to the EUCAST clinical breakpoints. One dilution step up or down is within the test variability. In the new EUCAST guidelines [28], the MIC values for (orally administered) fosfomycin for uncomplicated UTIs with *E. coli* were reduced from 32 to 8 mg/L. All *E. coli* specimens were correctly classified with the highest MIC value: 24 mg/L. Therefore, an adjustment to the antibiotic concentration in the assay has to be made to comply with the new guidelines. The results for cefuroxime showed 3 major (false-susceptible) and 13 minor errors, with *E. coli* most frequently misclassified. Possible reasons for the misclassification could be an inoculum effect described in the literature [29,30,31]. Therefore, a different number of bacteria used for the MALDI AST caused by, for example, residual leukocytes, could have an impact on the effectiveness of the antibiotic used in the assay. Another reason could be that exposure to beta-lactam antibiotics usually leads to an initial elongation of the bacteria, while still producing proteins, which are then detected by the MALDI-TOF MS [32]. This can lead to a false-resistant phenotype in the MALDI AST.

In general, possible factors contributing to major and minor errors in MALDI AST results are likely to include the incubation time, the number of bacteria used, and the medium used. We observed antibiotic-specific deviations in the MALDI AST results from the gold standard (the culture-based AST determination) of varying magnitude. It is thus necessary to individually vary the approach for each antibiotic to achieve a valid result. However, this leads to a more complex test execution at the expense of the workload and the time to result. Further improvement was not within the scope of this proof-of-concept study, so future studies will have to address this issue.

Mixed cultures from urine samples can occur because of contamination, colonization of the urinary tract, or a real polymicrobial infection. As previously described, this study failed to consistently detect resistant bacteria in a polymicrobial sample. For this reason, performing the MALDI AST with two different bacteria at the same time is not recommended. Since the MALDI AST is a rapid AST method, and the presence of more than one *Enterobacterales* cannot be ruled out a priori, we strongly recommend performing a conventional culture and conventional phenotypic AST in parallel to obtain definitive results.

The MALDI AST has been evaluated for positive blood cultures [33,34,35]. Because of previous incubation in positive blood cultures, bacteria showed good growth properties at the start of these MALDI ASTs. Therefore, direct comparisons with AST results obtained directly from patient urine samples are difficult. Jung et al. [33] and Axelsson et al. [34] reported no wrong classifications for cefotaxime in *E. coli* and *Klebsiella pneumoniae* isolates, even when the incubation time was reduced to 90 min. In contrast, Sauget et al. [35] could not confirm these encouraging results with a shortened incubation time, as was the case in the present study. Jung et al. reported similar problems with piperacillin–tazobactam. For ciprofloxacin, Axelsson et al. [34] found AST results with major and minor errors, whereas Jung et al. [33] correctly classified all isolates.

As with all AST methods applied directly to patient urine samples, a preselection of urine samples must be performed, as many do not show cultural growth and are, therefore, not suitable for direct ASTs.

A short incubation time of only 2.5 h was chosen for this study to provide same-day results for the most common UTI-causing bacteria, thereby limiting the AST results to fast-growing bacteria such as *Enterobacterales*. However, with a rapid MALDI AST, the incubation time is too short to differentiate between susceptible and resistant isolates of slower-growing *Pseudomonas aeruginosa* and most Gram-positive bacteria [36]. Adjustments to the protocol for these rarer UTI cases would come at the expense of the time to result but may be appropriate in settings with a high prevalence of these bacteria. 

In the present study, 24 samples failed to produce MALDI AST results despite the presence of *Enterobacterales* in sufficient quantity and having no inhibitory substances. Interestingly, these samples were characterized by high WBC counts and relatively low bacteria counts, which might have affected bacterial growth in the MALDI AST assay. To reduce the leucocyte count, adjusting the washing protocol might be helpful [37] to lower both the rate of unsuccessful MALDI ASTs and the general background noise in the MALDI-TOF MS caused by leucocyte proteins. As with AST methods in general [22], using the MALDI AST on urine samples with polymicrobial infections is a definite concern. The resistant isolates do not always prevail even when bacteria counts are equal to the susceptible isolates.

Because of the need for extensive equipment and expertise in the laboratory, MALDI AST assays are not suitable for point-of-care diagnostics. The MALDI AST requires approximately 2 h of hands-on time; because of the many different manual work steps, it is currently quite labor-intensive. Therefore, more automation and simplification of the test are needed to expand its application. A recent development in this regard is the direct-on-target microdroplet assay described by Idelevich et al. [38,39], which could be adapted to MALDI ASTs on urine samples. Further panel-based developments of the MALDI AST are desirable to pave the way for broader applications in clinical microbiology laboratories.

Since the MALDI-TOF MS is available in almost all microbiological laboratories, this AST method could be widely applied. As no extra equipment is required, the cost of the assay is expected to be rather low. In addition, bacterial identification can be performed simultaneously with an AST, providing important information for interpreting the results according to EUCAST guidelines. In conclusion, the MALDI AST is a suitable tool for detecting antibiotic resistance directly in patient urine samples in a much shorter time than conventional AST methods, thus providing same-day results to the physician.

## 4. Materials and Methods

All urine samples used in this study were retained samples from inpatients from a university hospital collected over a 3-month period. Therefore, many samples were taken from urinary catheters, and the pathogen spectrum differs slightly from what would be expected in outpatients. All samples were processed as anonymized samples within 12 h of collection, as no significant change in the bacterial count was to be expected in urine samples with stabilizers at this point [40,41].

Since many of the submitted urine samples showed no bacterial growth, it was necessary to preselect urine samples suitable for rapid ASTs using the MS ASTRA assay to identify urine samples with growing bacteria, particularly those with *Enterobacterales*. Therefore, urine samples were either analyzed with Cobas 9 manual urine dipstick tests (F. Hoffmann-La Roche AG; Basel, Switzerland) or a UF 5000 flow cytometer (Sysmex Deutschland GmbH; Norderstedt, Germany). Nitrite detected in the urine dipstick tests was used as a surrogate marker for *Enterobacterales.* The UF 5000 flow cytometer uses a correlate of microscopic Gram-staining (Gram-negative flag) to detect Gram-negative bacteria. In addition, pyuria (leukocyte esterase or leucocyte counts) was used to ensure the clinical relevance of the found pathogens. 

For preliminary testing, known susceptible and resistant Gram-negative bacteria were tested in an artificial urine medium [42] with antibiotic concentrations for the assay based on EUCAST clinical breakpoints [15]. Isolates of the species *E. coli*, *Klebsiella pneumoniae*, *Proteus mirabilis*, and *Serratia marcescens* were used in these preliminary tests. The antibiotics used in this study were commonly used antibiotics for UTIs, and most of them are recommended in the guidelines on urological infections from the European Association of Urology (EAU) [43] as first-line therapies. These include cotrimoxazole (Ratiopharm GmbH; Ulm, Germany) (concentration in the assay, 8 mg/L); fosfomycin (INFECTOPHARMA Arzneimittel und Consilium GmbH; Heppenheim, Germany) (64 mg/L); meropenem (Hikma Pharma GmbH; Planegg, Germany) (8 mg/L); nitrofurantoin (Sigma-Aldrich Co.; St. Louis, MO, USA) (64 mg/L); ciprofloxacin (0.5 mg/L); and cefuroxime (32 mg/L) (both Fresenius Kabi Deutschland GmbH; Langenhagen, Germany). If necessary, adjustments were made by gradually increasing the antibiotic concentration until reliable discrimination between susceptible and resistant specimens according to the respective EUCAST guidelines was possible.

After selecting suitable urine, 1 mL of urine was transferred to a 1.5 mL Eppendorf tube and centrifuged at 4 °C and 4300 rpm for 5 min. The supernatant was discarded, and the resulting pellet was resuspended with 1 mL Aqua dem. The sample was then centrifuged at 14,000 rpm for 2 min. This washing procedure was repeated once more. The pellet was then resuspended in approximately 50 µL of the supernatant and 5 µL was added to 1400 µL of AST medium (Becton Dickinson GmbH; Heidelberg, Germany). In total, 1000 µL of this was needed to determine the OD_600_ and calculate the dilution for a desired OD_600_ of 0.01. For each preparation, the antibiotic being tested and 200 µL of the diluted bacterial suspension was added to a 1.5 mL Eppendorf tube. The antibiotic was added based on the preliminary tests. In addition, a growth control of 200 µL was prepared. The preparations were then incubated for 2.5 h at 37 °C and 970 rpm in a shaking incubator. 

After the incubation, the entire 200 µL preparation was applied to a 96-well filter plate, centrifuged for 3 min at 4000 rpm and 20 °C, and the flow-through was discarded. The samples were then washed with 50 µL of HPLC-grade water (Carl Roth GmbH + Co. KG; Karlsruhe, Germany), centrifuged again, and the flow-through was discarded again. To lyse the bacteria, 10 µL of 70% formic acid (Sigma-Aldrich Co.; St. Louis, MI, USA) and 10 µL of acetonitrile (J.T. Baker, Thermo Fisher Scientific Inc.; Waltham, MA, USA) dissolved in Internal ASTRA Standard (Bruker Daltonik GmbH; Bremen, Germany) were added to each batch. After another centrifugation under the same conditions, the flow-through was collected in a fresh 96-well plate and used for measurement in the MALDI. To do this, 1 µL of flow-through was applied to each spot on a polished steel target plate. We used 4 spots per batch to allow the analysis software to average and be less affected by outliers. Additionally, after drying, 1 µL of HCCA (alpha-cyano-4-hydroxycinnamic acid from Bruker Daltonik GmbH; Bremen, Germany) was added as a matrix.

After drying the matrix, the target was placed in the MALDI-TOF, whereupon the MALDI-TOF MS measurement was performed and the measurement spectra were generated after the vacuum was established. Masses ranging from 2000 to 20,000 Da were measured using the same settings routinely used for pathogen identification.

Using the MALDI Biotyper MS ASTRA Prototype Software (Bruker Daltonik GmbH; Bremen, Germany), it was possible to automatically evaluate the data of the MALDI-TOF MS ASTRA assay. This software has been used successfully for blood cultures in our laboratory [33]. The prototype software was written in the free programming language R [44]. To analyze the spectra generated by the MALDI-TOF MS, the baseline was subtracted, and the peaks were normalized to the highest intensity value. These relative intensities between 0 and 1 were divided into 100 equally spaced regions (spacing, 0.01). The peaks above each threshold were counted and plotted against each other. From this, the software then calculated an area under the curve (AUC) for each spectrum [14,44,45]. This value reflects the growth of the bacteria in the preparation. From these values, box plots were generated for the four measurements per sample. A result above the threshold value of the AUC of 0.05 indicated growth in the tested sample. Below the threshold value, insufficient bacterial protein was detected. If the limit value of 0.05 was not exceeded in the preparation without an antibiotic, the sample could not be evaluated. If the limit value was not exceeded in the preparation with an antibiotic, the bacterium was classified as susceptible to this antibiotic. Since the AUC was sometimes incorrectly overestimated by the software because of background noise, the spectra were checked visually and corrected if necessary.

**Figure 4:**
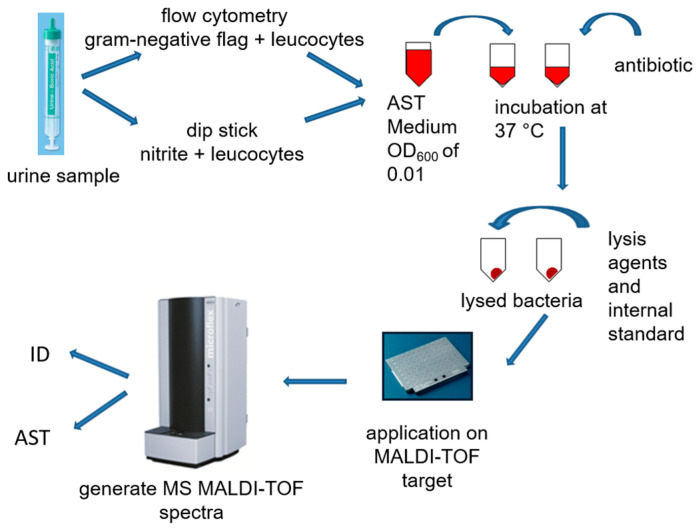
Workflow of the matrix-assisted laser desorption/ionization antimicrobial susceptibility testing (MALDI AST) used in this study. (AST: antibiotic susceptibility testing; ID: identification; MALDI-TOF: matrix-assisted laser desorption/ionization time-of-flight).

The test procedure described is shown schematically in Figure 4.

As a reference method to compare the results from the MALDI AST to a conventional AST, MIC test strips (Liofilchem S.r.l., Roseto degli Abruzzi (TE), Italy) were used. Subcultures were applied to Mueller-Hinton II agar plates (Becton Dickinson, Heidelberg, Germany) according to EUCAST guidelines [46], and the MIC values were determined according to the manufacturer´s instructions.

## 5. Conclusions

In conclusion, the MALDI AST is a suitable tool for detecting antibiotic resistance directly from patient urine samples in a much shorter time than conventional AST methods and can provide same-day results to physicians.

## Figures and Tables

**Table 1:** List of bacterial species found in the urine samples.

Bacterial Species	Number of Isolates from Monomicrobial Urines	Number of Isolates from Polymicrobial Urines
*E. coli*	66	6
*Klebsiella pneumoniae*	7	7
*Proteus mirabilis*	6	1
*Enterobacter cloacae*	4	2
*Klebsiella aerogenes*	3	-
*Klebsiella oxytoca*	3	2
*Serratia marcescens*	2	1
*Citrobacter freundii*	1	-
*Citrobacter koseri*	1	-
*Morganella morganii*	1	1
*Serratia liquefaciens*	1	-
*Proteus vulgaris*	-	1
Overall	95	21

**Table 2:** Overview of the agreement and the major (MEs) and minor errors (MiEs) between the conventional AST and the MALDI AST for the antibiotics used in this study.

	Tested Isolates	Agreement	ME	%	MiE	%
Cefuroxime	79	79.8%	3	3.8%	13	16.5%
Ciprofloxacin	95	95.8%	0	0.0%	4	4.2%
Cotrimoxazole	92	93.5%	0	0.0%	6	6.5%
Fosfomycin	66	100.0%	0	0.0%	0	0.0%
Meropenem	95	100.0%	0	0.0%	0	0.0%
Nitrofurantoin	66	100.0%	0	0.0%	0	0.0%
Overall	493	94.7%	3		22	

## Data Availability

The data presented in this study are available in the article and the Supplementary Materials.

## Supplementary Materials

The following supporting information can be downloaded at https://www.mdpi.com/article/10.3390/antibiotics12061042/s1: Figure S1: cefuroxime results. Figure S2: cotrimoxazole results. Figure S3: fosfomycin results for *E. coli*. Figure S4: meropenem results. Figure S5: nitrofurantoin results for *E. coli*. Table S6: overview of all urine samples with a MALDI AST and the corresponding bacteria found. Table S7: overview of urine samples with polymicrobial growth.
