# Supplementary material for: Rapid Antibiotic Susceptibility Testing of Gram-Negative Bacteria Directly from Urine Samples of UTI Patients Using MALDI-TOF MS
Source: Antibiotics (Basel). 2023 Jun 12;12(6):1042. doi: 10.3390/antibiotics12061042 (PMC10295066; doi:10.3390/antibiotics12061042)
Supplement: Supplementary file 1 [file antibiotics-12-01042-s001.zip › antibiotics-2394652-supplementary.pdf]

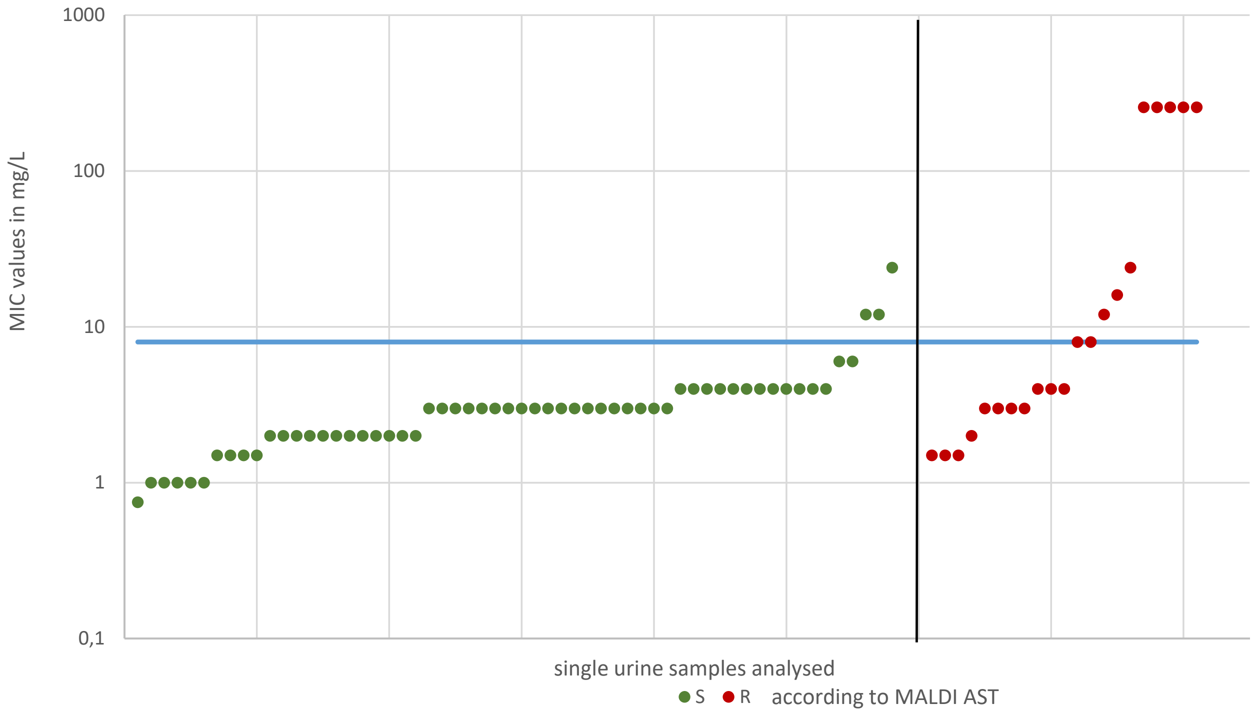

Figure S1. Cefuroxime results: Each bacteria sample from a patient´s urine (on the X-axis) is assigned a respective MIC value from the MIC test strip (on the Y-axis). Each dot represents one sample. All samples are grouped by MALDI AST results (S, R) and ascending MIC values. Green dots indicate a susceptible result, and red dots indicate a resistant result in the MALDI AST assay (separated by the black line). The MIC value breakpoints is 8 mg/L (blue lines).

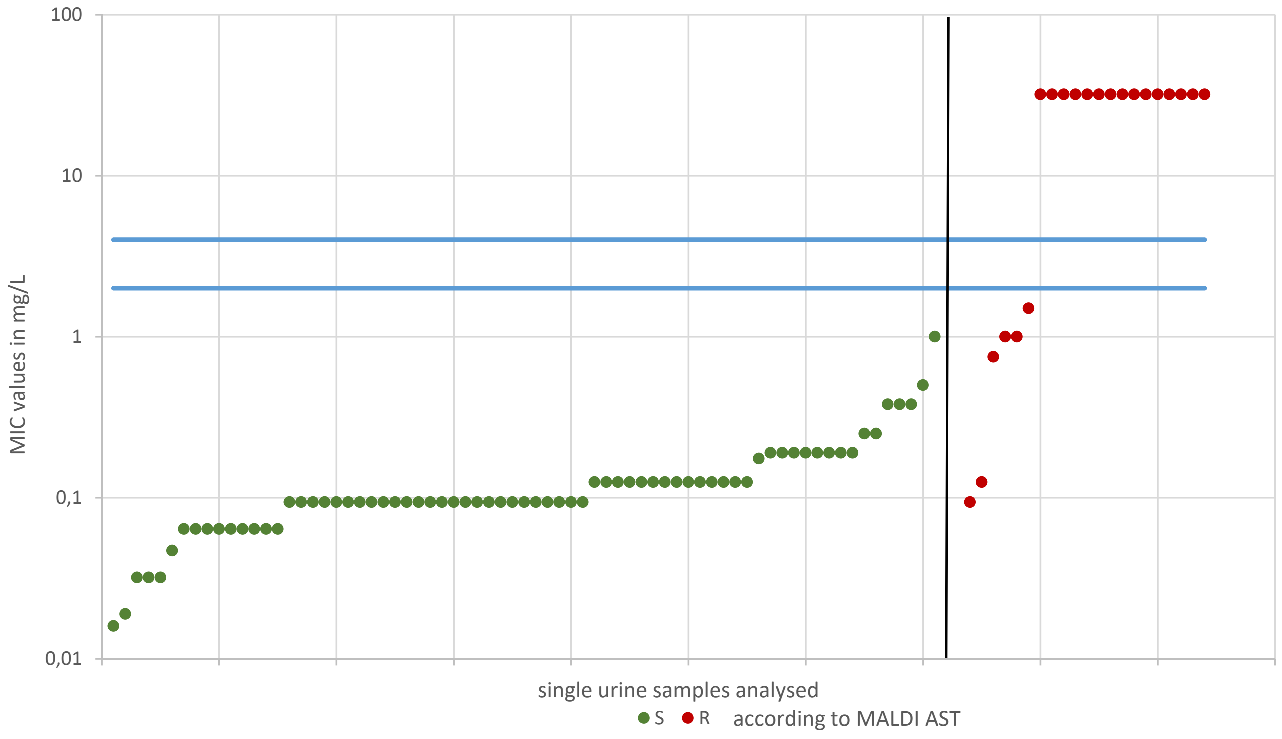

Figure S 2. Cotrimoxazole results: Each bacteria sample from a patient´s urine (on the X-axis) is assigned a respective MIC value from the MIC test strip (on the Y-axis). Each dot represents one sample. All samples are grouped by MALDI AST results (S, R) and ascending MIC values. Green dots indicate a susceptible result, and red dots indicate a resistant result in the MALDI AST assay (separated by the black line). The MIC value breakpoints are 2 mg/L and 8 mg/L (blue lines).

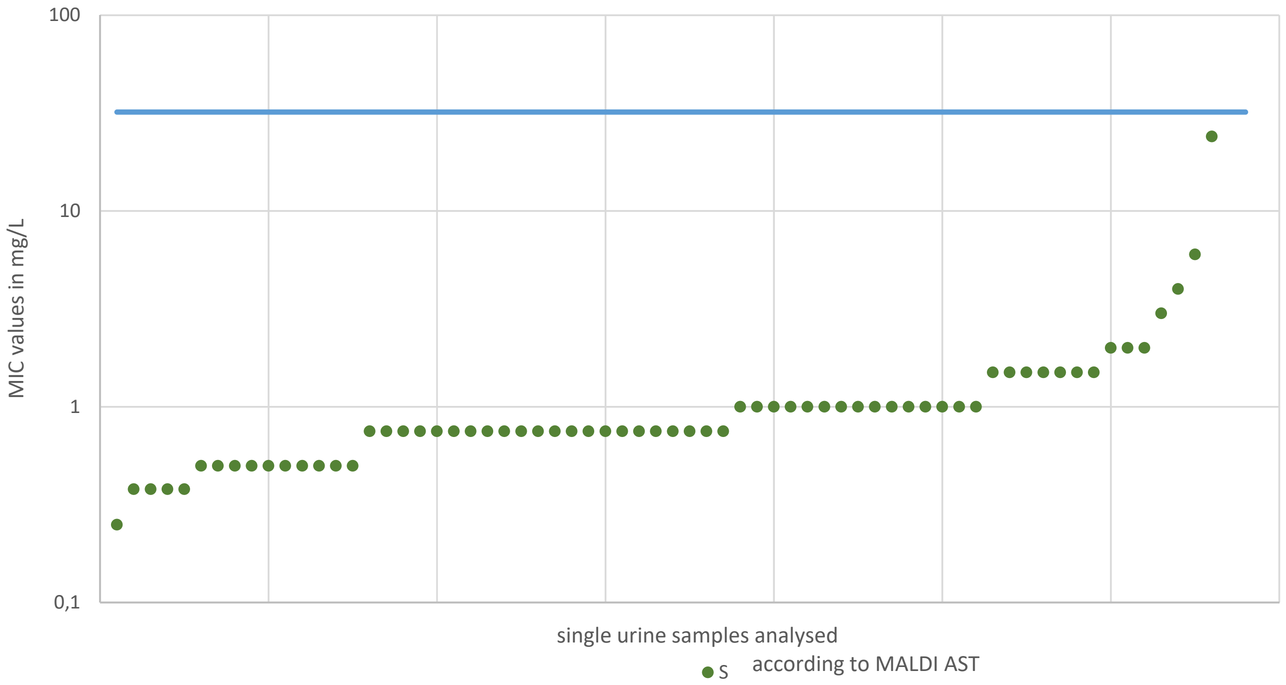

Figure S 3. Fosfomycin results: Each bacteria sample from a patient´s urine (on the X-axis) is assigned a respective MIC value from the MIC test strip (on the Y-axis). Each dot represents one sample. All samples are grouped by MALDI AST results (S, R) and ascending MIC values. Green dots indicate a susceptible result, and red dots indicate a resistant result in the MALDI AST assay (separated by the black line). The MIC value breakpoint is 32 mg/L (blue line).

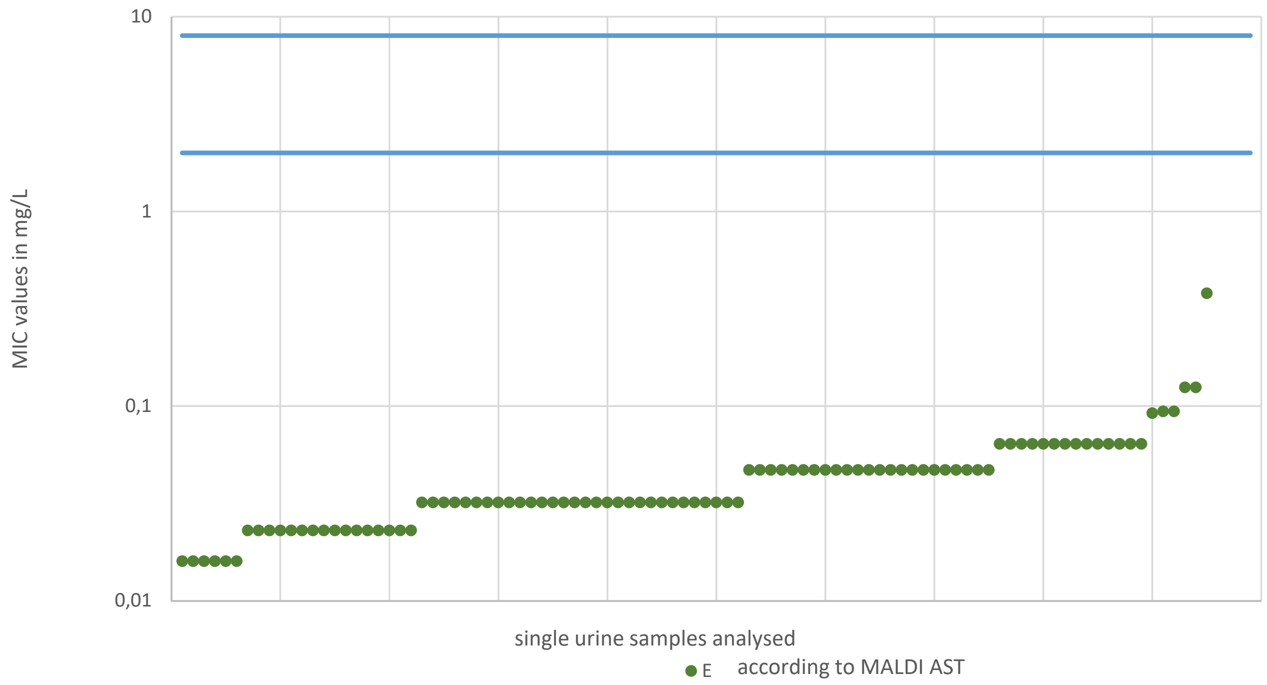

Figure S 4. Meropenem results: Each bacteria sample from a patient´s urine (on the X-axis) is assigned a respective MIC value from the MIC test strip (on the Y-axis). Each dot represents one sample. All samples are grouped by MALDI AST results (S, R) and ascending MIC values. Green dots indicate a susceptible result, and red dots indicate a resistant result in the MALDI AST assay (separated by the black line). The MIC values breakpoints are 2 mg/L and 8 mg/L (blue lines).

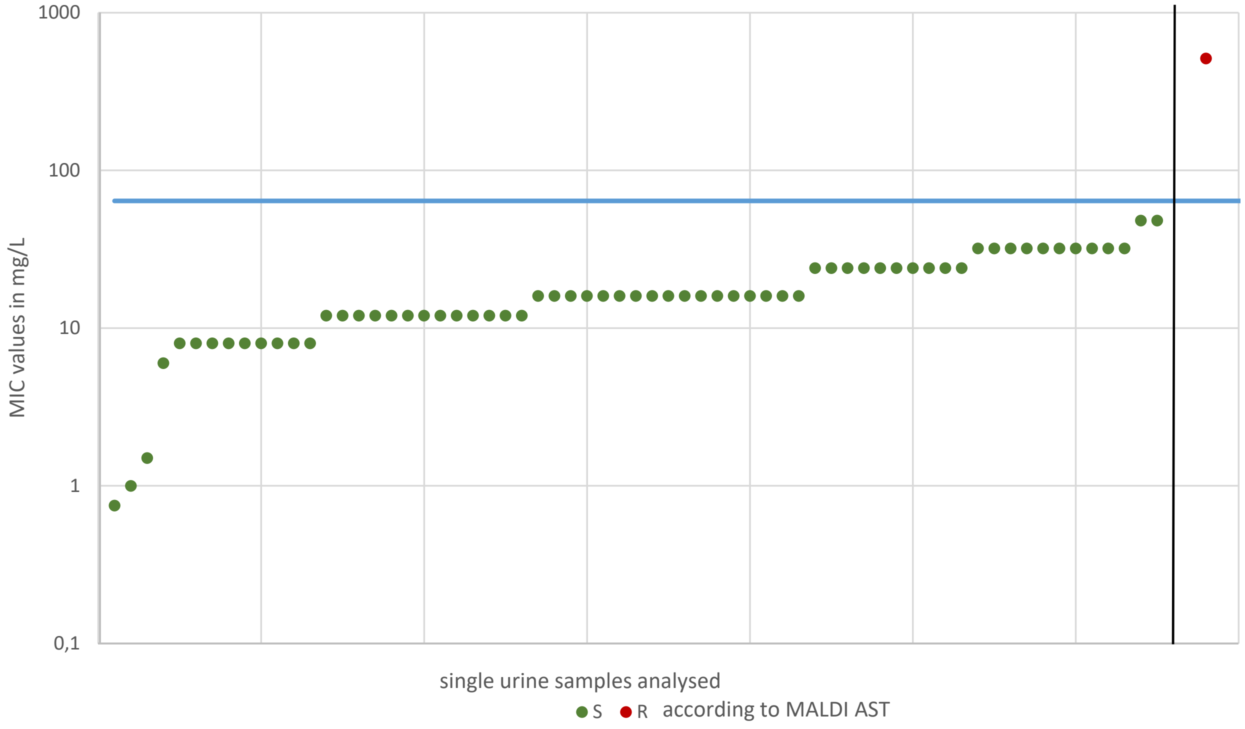

Figure S 5. Nitrofurantoin results: Each bacteria sample from a patient´s urine (on the X-axis) is assigned a respective MIC value from the MIC test strip (on the Y-axis). Each dot represents one sample. All samples are grouped by MALDI AST results (S, R) and ascending MIC values. Green dots indicate a susceptible result, and red dots indicate a resistant result in the MALDI AST assay (separated by the black line). The MIC value breakpoint is 64 mg/L (blue line).

CFU/ml	uropathogens	MALDI AST						Conventional AST					
		CIP	CEF	STX	FF	MEM	NIF	CIP	CEF	STX	FF	MEM	NIF
10^5	*Citrobacter freundii*	E	-	E	-	E	-	0.012	-	0.19	-	0.023	-
10^5	*Citrobacter koseri*	E	-	E	-	E	-	0.012	-	0.19	-	0.032	-
10^5	*E. coli*	E	E	E	E	E	E	0.008	0.75	0.094	1.5	0.016	24
10^5	*E. coli*	E	E	E	E	E	E	0.008	1.5	0.094	0.75	0.016	16
10^5	*E. coli*	E	E	E	E	E	E	0.06	1.5	0.094	1	0.023	12
10^5	*E. coli*	E	E	E	E	E	E	0.008	2	0.032	0.38	0.016	8
10^5	*E. coli*	E	E	E	E	E	E	0.012	2	0.064	0.5	0.032	16
10^5	*E. coli*	E	E	E	E	E	E	0.008	2	0.064	0.75	0.047	8
10^5	*E. coli*	E	E	E	E	E	E	0.008	2	0.064	3	0.064	32
10^5	*E. coli*	E	E	E	E	E	E	0.012	2	0.094	0.75	0.016	0.75
10^5	*E. coli*	E	E	E	E	E	E	0.016	2	0.094	0.75	0.023	16
10^5	*E. coli*	E	E	E	E	E	E	0.19	2	0.094	1	0.032	8
10^5	*E. coli*	E	E	E	E	E	E	0.012	2	0.125	0.38	0.023	16
10^5	*E. coli*	E	E	E	E	E	E	0.012	2	0.125	0.5	0.047	12
10^5	*E. coli*	E	E	R	E	E	E	0.125	2	> 32	0.5	0.092	32
10^5	*E. coli*	E	E	E	E	E	E	0.016	3	0.032	1	0.023	1.5
10^5	*E. coli*	E	E	E	E	E	E	0.012	3	0.064	1	0.023	16
10^5	*E. coli*	E	E	E	E	E	E	0.006	3	0.064	24	0.023	8
10^5	*E. coli*	E	E	E	E	E	E	0.008	3	0.094	0.38	0.023	12
10^5	*E. coli*	E	E	E	E	E	E	0.012	3	0.094	0.25	0.032	24
10^5	*E. coli*	E	E	E	E	E	E	0.008	3	0.094	0.75	0.032	32
10^5	*E. coli*	E	E	E	E	E	E	0.012	3	0.094	1.5	0.032	48
10^5	*E. coli*	E	E	E	E	E	E	0.012	3	0.125	0.5	0.032	16
10^5	*E. coli*	E	E	E	E	E	E	0.008	3	0.125	1	0.032	24
10^5	*E. coli*	E	E	E	E	E	E	0.008	3	0.175	0.75	0.047	8
10^5	*E. coli*	E	E	R	E	E	E	0.006	3	1	2	0.032	6
10^5	*E. coli*	E	E	R	E	E	E	0.008	3	1.5	1	0.032	24
10^5	*E. coli*	R	E	R	E	E	E	6	3	> 32	0.5	0.016	32

CFU/ml	uropathogens	MALDI AST						Conventional AST					
		CIP	CEF	STX	FF	MEM	NIF	CIP	CEF	STX	FF	MEM	NIF
10^5	*E. coli*	E	E	R	E	E	E	0.047	3	> 32	1	0.023	12
10^5	*E. coli*	E	E	E	E	E	E	0.012	4	0.064	1.5	0.047	16
10^5	*E. coli*	E	E	E	E	E	E	0.008	4	0.094	0.75	0.032	16
10^5	*E. coli*	E	E	E	E	E	E	0.008	4	0.094	1	0.032	32
10^5	*E. coli*	E	E	E	E	E	E	0.008	4	0.094	0.5	0.032	16
10^5	*E. coli*	E	E	E	E	E	E	0.012	4	0.125	0.75	0.032	32
10^5	*E. coli*	E	E	E	E	E	E	0.012	4	0.38	4	0.023	24
10^5	*E. coli*	E	E	E	E	E	E	0.016	4	0.125	1	0.032	32
10^5	*E. coli*	R	E	R	E	E	E	0.19	4	0.75	1	0.047	12
10^5	*E. coli*	E	E	R	E	E	E	0.008	4	> 32	0.75	0.032	24
10^5	*E. coli*	R	E	R	E	E	E	6	4	> 32	0.75	0.047	32
10^5	*E. coli*	E	E	E	E	E	E	0.008	6	0.19	0.75	0.032	16
10^5	*E. coli*	R	E	R	E	E	E	> 32	24	> 32	0.75	0.047	16
10^5	*E. coli*	E	E	E	E	E	E	0.006	2	0.094	0.38	0.032	12
10^5	*E. coli*	E	E	E	E	E	E	0.023	12	0.094	1.5	0.047	12
10^5	*E. coli*	R	E	R	E	E	E	> 32	12	> 32	1.5	0.023	8
10^5	*E. coli*	E	E	E	E	E	E	0.008	3	0.125	0.75	0.032	16
10^4	*E. coli*	E	E	E	E	E	E	0.012	4	0.016	1	0.032	16
10^5	*E. coli*	E	E	E	E	E	E	0.008	6	0.25	1	0.047	24
10^5	*E. coli*	R	R	R	E	E	E	> 32	1.5	> 32	0.75	0.047	16
10^5	*E. coli*	R	R	E	E	E	E	4	3	0.094	0.5	0.047	16
10^5	*E. coli*	E	R	R	E	E	E	0.008	3	0.125	6	0.047	48

CFU/ml	uropathogens	MALDI AST						Conventional AST					
		CIP	CEF	STX	FF	MEM	NIF	CIP	CEF	STX	FF	MEM	NIF
10^5	*E. coli*	R	R	E	E	E	E	0.19	4	0.064	0.5	0.032	12
10^5	*E. coli*	E	R	E	E	E	E	0.012	4	0.125	0.75	0.032	16
10^5	*E. coli*	R	R	R	E	E	E	> 32	12	> 32	0.5	0.032	12
10^5	*E. coli*	E	R	E	E	E	E	0.032	24	0.094	1.5	0.032	8
10^5	*E. coli*	E	R	E	E	E	E	0.016	8	0.064	2	0.064	32
10^5	*E. coli*	R	R	E	E	E	E	> 32	8	0.19	1	0.032	32
10^5	*E. coli*	R	R	E	E	E	E	> 32	> 256	0.094	1	0.023	8
10^5	*E. coli*	R	R	R	E	E	E	> 32	> 256	> 32	0.75	0.047	8
10^5	*E. coli*	E	-	-	E	E	R	0.047	-	-	0.75	0.064	> 512
10^5	*E. coli*	E	-	-	E	E	E	0.008	-	-	0.75	0.047	12
10^5	*E. coli*	E	E	E	E	E	E	0.008	3	0.125	0.75	0.023	12
10^5	*E. coli*	E	E	E	E	E	E	0.012	3	0.38	1.5	0.016	24
10^5	*E. coli*	E	E	E	E	E	E	0.008	4	0.125	1	0.047	24
10^5	*E. coli*	E	E	E	E	E	E	0.012	3	0.094	0.5	0.047	16
10^5	*E. coli*	E	R	E	E	E	E	0.006	2	0.032	0.75	0.023	1
10^5	*E. coli*	E	R	E	E	E	E	0.008	4	1	0.75	0.047	12
10^5	*E. coli*	R	R	E	E	E	E	0.064	> 256	0.094	0.75	0.032	12
10^5	*E. coli*	R	R	R	E	E	E	> 32	> 256	> 32	2	0.064	24
10^5	*Enterobacter cloacae*	E	-	E	-	E	-	0.008	-	0.064	-	0.032	-
10^5	*Enterobacter cloacae*	E	-	R	-	E	-	0.006	-	0.094	-	0.032	-
10^5	*Enterobacter cloacae*	E	-	E	-	E	-	0.008	-	0.125	-	0.047	-
10^5	*Enterobacter cloacae*	E	-	E	-	E	--	0.016	-	0.125	-	0.047	-
10^5	*Klebsiella aerogenes*	E	-	E	-	E	-	0.016	-	0.094	-	0.047	-
10^5	*Klebsiella aerogenes*	E	-	E	-	E	-	0.023	-	0.125	-	0.064	-
10^5	*Klebsiella aerogenes*	E	-	E	-	E	-	0.016	-	0.19	-	0.064	-

CFU/ml	uropathogens	MALDI AST						Conventional AST					
		CIP	CEF	STX	FF	MEM	NIF	CIP	CEF	STX	FF	MEM	NIF
10^5	*Klebsiella oxytoca*	E	R	R	-	E	-	0.125	1.5	> 32	-	0.032	-
10^5	*Klebsiella oxytoca*	R	R	E	-	E	-	0.38	3	0.094	-	0.047	-
10^5	*Klebsiella oxytoca*	E	R	E	-	E	-	0.008	3	0.094	-	0.064	-
10^5	*Klebsiella pneumoniae*	E	E	E	-	E	-	0.016	1.5	0.047	-	0.047	-
10^5	*Klebsiella pneumoniae*	E	E	E	-	E	-	0.023	1.5	0.19	-	0.064	-
10^5	*Klebsiella pneumoniae*	E	E	E	-	E	-	0.23	2	0.094	-	0.047	-
10^5	*Klebsiella pneumoniae*	E	E	R	-	E	-	0.023	3	> 32	-	0.047	-
10^5	*Klebsiella pneumoniae*	E	R	R	-	E	-	0.019	16	1	-	0.023	-
10^5	*Klebsiella pneumoniae*	R	R	R	-	E	-	> 32	> 256	> 32	-	0.064	-
10^5	*Klesbiella pneumoniae*	E	-	-	-	E	-	0.25	-	-	-	0.064	-
10^5	*Morganella morganii*	E	-	E	-	E	-	0.008	-	0.094	-	0.125	-
10^5	*Proteus mirabilis*	E	E	E	-	E	-	0.016	1	0.094	-	0.125	-
10^5	*Proteus mirabilis*	E	E	E	-	E	-	0.094	1	0.5	-	0.032	-
10^5	*Proteus mirabilis*	E	E	R	-	E	-	0.032	1	> 32	-	0.064	-
10^5	*Proteus mirabilis*	E	R	E	-	E	-	0.016	1.5	0.25	-	0.38	-
10^5	*Proteus mirabilis*	E	E	E	-	E	-	0.047	1	0.38	-	0.094	-
10^5	*Proteus mirablis*	E	E	E	-	E	-	0.016	1	0.019	-	0.064	-
10^5	*Serratia liquefacines*	E	-	E	-	E	-	0.023	-	0.19	-	0.023	-
10^5	*Serratia marcenscens*	E	-	E	-	E	-	0.125	-	0.125	-	0.064	-
10^5	*Serratia marcescens*	E	-	E	-	E	-	0.032	-	0.19	-	0.094	-

Table S 6. Overview of all urine samples with a MALDI AST and the corresponding bacteria found: All results for antibiotics with breakpoint according to EUCAST were included in this table. The MIC values for conventional AST are in mg/L. CIP: ciprofloxacin, CEF: cefuroxime, STX: cotrimoxazole, FF: fosfomycin, MEM: meropenem, NIF: nitrofurantoin

CFU/ml		CIP	CEF	STX	FF	MEM	NIF
	MS-ASTRA assay	S	R	S	S	S	S
10^5	*Klebsiella pneumoniae*	S	S	S	-	S	-
10^5	*E. coli*	S	S	S	S	S	S
	MS-ASTRA assay	S	R	S	S	S	S
10^5	*E. coli*	S	S	S	S	S	S
10^5	*Klebsiella pneumoniae*	S	S	S	-	S	-
10^5	*Proteus vulgaris*	S	-	S	-	S	-
	MS-ASTRA assay	S	S	S	-	S	S
10^5	*Klebsiella pneumoniae*	S	S	S	-	S	S
10^4	*Proteus mirabils*	R	S	R	-	S	-
	MS-ASTRA assay	S	R	S	S	S	S
10^5	*E. coli*	S	S	S	R	S	S
10^5	*Klebsiella pneumoniae*	S	S	S	-	S	-
	MS-ASTRA assay	S	R	S	-	S	-
10^5	*Enterobacter cloacae*	S	-	S	-	S	-
10^5	*Klebsiella oxytoca*	S	S	S	-	S	-

CFU/ml		CIP	CEF	STX	FF	MEM	NIF
	MS-ASTRA assay	S	S	R	S	S	S
10^5	*E. coli*	S	S	R	S	S	S
10^5	*Klebsiella pneumoniae*	R	R	R	-	S	-
	MS-ASTRA assay	S	R	S	-	S	-
10^5	*Klebsiella oxytoca*	S	S	S	-	S	-
10^5	*Enterobacter cloacae*	S	-	S	-	S	-
	MS-ASTRA assay	S	R	S	S	S	S
10^5	*E. coli I*	S	S	S	S	S	S
10^5	*E. coli II*	S	S	S	S	S	S
	MS-ASTRA assay	R	R	S	-	S	-
10^5	*Klebsiella pneumoniae I*	S	S	S	-	S	-
10^5	*Klebsiella pneumoniae II*	S	R	S	-	S	-
	MS-ASTRA assay	S	-	S	-	S	-
10^5	*Serratia marcescens*	S	-	S	-	S	-
10^5	*Morganella morganii*	S	-	S	-	S	-

Table S 7. Overview of urine samples with polymicrobial growth: All results for antibiotics with breakpoint according to EUCAST were included in this table. CIP: ciprofloxacin, CEF: cefuroxime, STX: cotrimoxazole, FF: fosfomycin, MEM: meropenem, NIF: nitrofurantoin
